# Effect of Concurrent Resistance Training on Lower Body Strength, Leg Kick Swimming, and Sport-Specific Performance in Competitive Swimmers

**DOI:** 10.3390/biology11020299

**Published:** 2022-02-12

**Authors:** Sofiene Amara, Tiago Manuel Barbosa, Oussama Gaied Chortane, Raouf Hammami, Ahmed Attia, Sabri Gaied Chortane, Roland van den Tillaar

**Affiliations:** 1 Higher Institute of Sport and Physical Education of Ksar-Said, University of La Manouba, Tunis 2010, Tunisia; oussama.gaeid@gmail.com (O.G.C.); raouf.cnmss@gmail.com (R.H.); attiaahmed3@yahoo.fr (A.A.); sabrigaied1@gmail.com (S.G.C.); 2 Research Unit (UR17JS01) Sports Performance, Health & Society, Higher Institute of Sport and Physical Education of Ksar Saîd, Universite de la Manouba, Tunis 2010, Tunisia; 3 Research Center in Sport, Health and Human Development, 5000-801 Vila Real, Portugal; barbosa@ipb.pt; 4 Department of Sports Sciences, Instituto Politécnico de Bragança, Campus Sta., 5301-856 Bragança, Portugal; 5 Research Laboratory: Education, Motor Skills, Sports and Health (LR19JS01), Higher Institute of Sport and Physical Education of Sfax, University of Sfax, Sfax 3029, Tunisia; 6 Laboratory of Cardio-Circulatory, Respiratory, Metabolic and Hormonal Adaptations to Muscular Exercise, Faculty of Medicine Ibn El Jazzar, Sousse 4002, Tunisia; 7 Department of Sport Sciences and Physical Education, Nord University, 7600 Levanger, Norway

**Keywords:** dry land training, one repetition maximum, back squat, water parachute, aquatic training, swimming performance

## Abstract

**Simple Summary:**

Resistance training in and out of the water aims to improve swimming performance. Previous studies have shown that dry land resistance training has positive effects on improving strength and therefore this could optimize swimming performance. The present study investigated the effect of 9 weeks of combined resistance training (aquatic and dry land resistance) on maximum lower body strength, leg kick, and swimming performance in competitive swimmers. The results demonstrated that 9 weeks of combined resistance training could improve the maximum lower body strength and leg kick swimming performance. These improvements can be the essential factors that subsequently positively affected swimming start and turn performance.

**Abstract:**

The present study investigated the effect of 9 weeks of combined resistance training (aquatic and dry land resistance) on maximum lower body strength, leg kick, and swimming performance in competitive swimmers. Twenty-two male national competitive swimmers were randomly assigned into two groups: experimental group (EG: age = 16.2 ± 0.3 years) or control group (CG: age = 16.3 ± 0.3 years). The EG performed a combined resistance training while the CG group completed their usual training. One repetition maximum (1RM) back squat, 30 m leg kick, and swimming performance (100 m front crawl, start and turn) were evaluated in pre and post test. The findings showed a significant increase in 1RM back squat (d = 1.90; 14.94 ± 1.32%) after 9 weeks of combined resistance training. In addition, ours results revealed a significant improvement in 30 m leg kick swimming (d = 2.11; 5.84 ± 0.16%) and in all swimming, start and turn performances (d = 1.83 to 2.77; 2.69 ± 0.18% to 15.14 ± 1.06%) in EG. All dependent variables remained unchanged in the CG. To sum up, 9 weeks of combined resistance training can improve the maximum lower body strength and leg kick swimming performance. These improvements can be the essential factors that subsequently positively affected swimming, start and turn performances. Combined resistance training is an effective training that can be incorporated by coaches and swimmers into their programs to improve strength, leg kick swimming, and, subsequently, swimming performance in competitive swimmers.

## 1. Introduction

Over the years, the 100 m race times have been improved probably due to better aquatic and dry land training programs [1,2]. Concurrent resistance training on dry land and in water showed to be an effective approach to improve swimming performance [1,3,4]. Amara et al. [1] noted that nine weeks of concurrent resistance training could improve maximal upper body strength (12.11 ± 1.79%) and sprint performance in front crawl (4.2 ± 0.2% to 7.1 ± 0.2%) in male competitive swimmers (age = 16.5 ± 0.30 years). Moreover, Lopes et al. [3] reported a significant improvement in sprint swimming performance (4.0% to 4.3%) after eight weeks of dry land strength combined with swimming training in university swimmers of national level (age = 20.55 ± 1.76 years). However, most studies focus upon increasing strength in the upper body, while improving the performance of the lower limbs is also an important factor in determining swimming performance [1,5]. Morouço et al. [5] showed that a relative contribution of leg kick was 29.7% for male swimmers and 33.4% for females. In the same context, Bartolomeu et al. [6] revealed that swim velocity with leg kick was 59% compared with full front crawl velocity in competitive swimmers (age = 14.20 ± 1.71 years). Furthermore, the start and turn are among the most important factors in determining swimming performance these days in competition settings. Morais et al. [7] showed that the start and the turn phases combined accounted for 31% to 32% of the final race time in the four swimming strokes. Notwithstanding, lower body strength and power are found to be two very important underlying factors determining the performance of start and turn in competitive swimmers [8,9]. Thus, it is necessary to know more about the effect of combined resistance training on maximum lower body strength, leg kick swimming, and swimming performance.

Lower limbs strength routines (e.g., countermovement jumps (CMJ), squat, and leg extension) are heavily used in several dry land swimming training programs [3,10]. For instance, Lopes et al. [3] showed that eight weeks of dry land training, including full squat and CMJ exercises, could improve CMJ performance (6.77%) in national competitive swimmers. Moreover, Sammoud et al. [10] revealed that 8 weeks of dry land training based on plyometric jump training (CMJ) could optimize the performance of CMJ (24.5%) and the performance of 25 m front crawl swimming (6.33%) in prepubertal female swimmers (age = 10.0 ± 0.6 years). These authors [3,10] showed that lower body dry land training optimized strength and swimming performance, but the information about the transfer of strength gain in water remains insufficient. Hence, it is necessary to understand the effect of dry land training on leg kick swimming performance.

Resistance training in water with aquatic equipment (e.g., water parachute) is also an effective approach to improve the propulsive force [1,11]. Gourgoulis et al. [11] noted that 11 weeks of training with water parachute improved the performance in the 50, 100, and 200 m front crawl (3.2% to 7.3%) in competitive swimmers. Amara et al. [1] reported that the 50 m front crawl performance was improved after 9 weeks of resistance training in water with water parachute (4.22 ± 0.18%) in young competitive swimmers. Amara et al. [1] also showed that an aquatic resistance training program can improve the performance in the 50 m front crawl arm-pull (7.1 ± 0.23%). The question arises here, could resistance training with this aquatic equipment improve leg kick performance? If so, how efficient is the transfer of dry land strength improvement into in-water leg kick performance?

Training in water with additional resistance by the water parachute is shown to be an effective training to improve swimming performance [11]. However, information about the effect of combined resistance training (additional aquatic resistance in and dry land resistance) on leg kick and swimming performance remains insufficient. Therefore, the objective of this study was to investigate the effect of lower body strength and additional aquatic resistance training (using water parachute) on strength, leg kick, and swimming performance in competitive swimmers. We hypothesized that improving lower body strength could improve leg kick performance and subsequently optimize swimming performance.

## 2. Materials and Methods

### 2.1. Experimental Approach to the Problem

The present study was applied to examine the effect of combined resistance training (aquatic and dry land resistance training) during a nine-week intervention period on maximum lower body strength (1RM back squat) and swimming performance (100 m front crawl, 30 m leg kick swimming, start and turn performance) compared to standard training. All swimmers participated in a week of familiarization for resistance training in dry land and in the water. All dependent variables were measured pre and post test. All dry land training and testing were performed in the weight room, and all swimming training and testing were performed in a 50 m indoor pool with 27.1 and 25.9 °C water and air temperatures (respectively) and 64% relative humidity. All swimmers were asked to avoid all other intensive activities and avoid all stimulating nutrients during the entire experimental period.

### 2.2. Subjects

Twenty-two male national competitive swimmers were randomly assigned into two groups: experimental group (EG: *n* = 11, age = 16.5 ± 0.3 years; height: 174 ± 9.80 cm; body mass = 72.7 ± 5.30 kg) or control group (CG: *n* = 11, age = 16.1 ± 0.3 years; height: 175 ± 9.70 cm body mass 73.6 ± 5.25 kg). An a priori power analysis (G * Power 3.1.9.3, Heinrich-Heine-Universität Düsseldorf, Düsseldorf, Germany) yielded a sample size of at least 9 swimmers per group to detect large effects (d = 1.29), assuming a power of 0.8 and alpha of 0.05. All subjects had three years of experience in resistance training and 5 years of experience in resistance aquatic training. The best time of the fastest swimmer in 100 m front crawl was 57.08 s. All swimmers were informed about all research-related risks and potential benefits. All subjects and parents read and signed written informed consent. This study was approved by an institutional review board of Higher Institute of Sport and Physical Education of Ksar Said, University of Manouba, Tunisia (Research Unit of Sports Performance, Health & Society, UR17JS01) and conducted according to the last declaration of Helsinki.

### 2.3. Procedures

**Figure 1:**
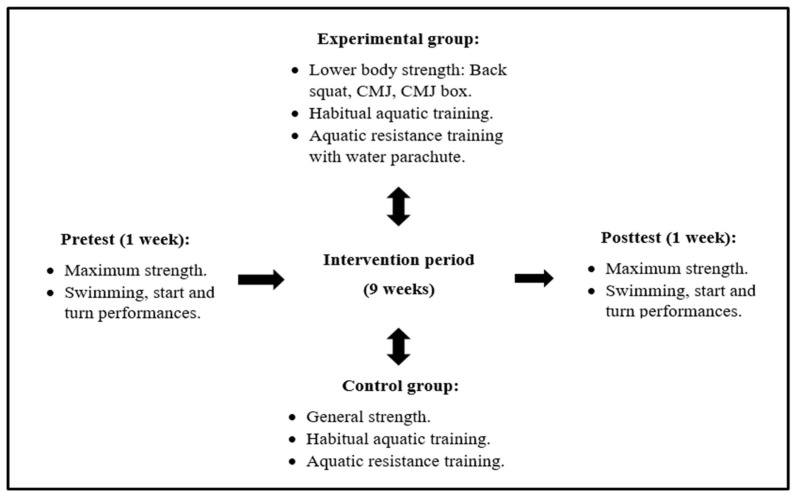
Overview of the study design. CMJ: countermovement jump; CMJ box: countermovement jump box.

This study was integrated into the preparatory phase of the winter competition (September to December). All swimmers were conditioned to training and have started the training season at the same time (3 months before the experimental protocol). The study design consisted of a pretest followed-up by 9 weeks of intervention and a one-week pos test (Figure 1).

#### 2.3.1. Aquatic Resistance Training

**Figure 2:**
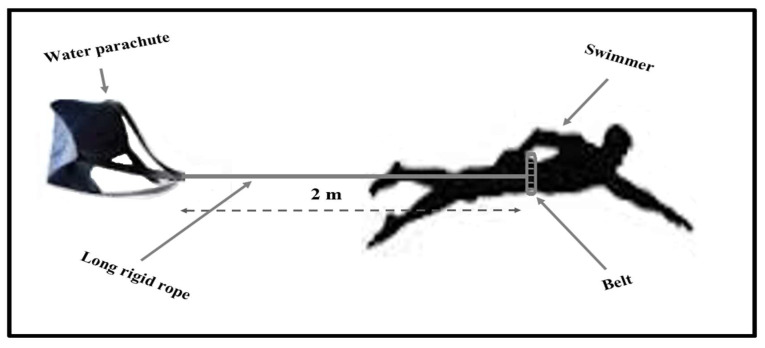
Set-up of water parachute during aquatic resistance training.

The swimming training program was composed of six sessions per week with an hourly volume between 90 and 120 min, and swimming distance between 4000 and 6000 m per session. All-out sets of specific resistance training using only a leg kick (4 sessions in a week) were included in both training programs (EG and CG) immediately after the warm-up (800 m of aerobic training (55% to 80% of maximum heart rate). On Monday and Thursday, swimmers completed 3 sets × 6 reps × 15 m with 60 s and 5 min of rest between repetitions and sets, respectively. On Tuesday and Friday, swimmers completed 2 sets × 4 reps × 25 m with 90 s and 5 min of rest between repetitions and sets, respectively [1,11]. To eliminate the effect of arm stroke, specific resistance training was conducted with the aid of a kickboard, which was held by both hands. The experimental group completed the specific aquatic training with additional resistance using a small water parachute (288 cm^2^). The water parachute was attached to the swimmer’s back by a 2 m-long rigid rope with a belt that was fasted around the swimmer’s waist [1] (Figure 2).

#### 2.3.2. Lower Body Strength Training

Two non-consecutive sessions of lower body strength per week (separated by 48 h) were set up. Two strength and conditioning coaches designed and conducted the dry land training. Each session started with a 20 min standard warm-up featuring aerobic exercises (ergocycle and treadmill) and two sets of 8 to 10 reps of back squat with lower load (20% to 30% 1RM back squat). Subsequently, subjects performed three lower body strength exercises, which were back squat, countermovement jump (CMJ), and CMJ box with moderate contraction velocity and complete motion angle. The back squat exercise was performed with an intensity between 60% and 80% of 1RM. The sets varied between 2 and 3 and repetitions between 6 and 10. A custom power rack and a standard Olympic weightlifting bar calibrated and certified (20 kg) were used to perform the back squat and to better control the technique of performing the exercise. Moreover, CMJ and CMJ box exercises consisted of 6 to 8 sets with 6 to 10 repetitions. The recovery between sets and exercises was fixed at 2 min (Table 1) [1,12,13]. The CG was invited to follow their usual training based on general body strength (2 sessions per week: 60 to 75 min per session). General body strength consisted of a general warm-up (cardiorespiratory adaptation and muscle stimulation), general strength exercises (pump-action, medicine ball throw, abdominal exercises), and muscle stretching.

#### 2.3.3. Maximum Lower Body Strength Test

On the first day of testing (at 10.00 am), subjects visited the weight room to determine the 1RM back squat test. The strength test was performed using a custom power rack and a standard Olympic weight lifting bar calibrated and certified (20 kg). Two strength and conditioning coaches controlled the execution of the exercises and conducted all measurements of the maximum back squat strength test.

All subjects completed a warm-up on an ergocycle for 3 min, followed-up by 5 min of overall static stretching. Thereafter, subjects performed 1 set of 8 reps and 1 set of 3 reps at 50% and 70% of their estimated 1RM back squat, respectively. The load was gradually increased (10% to 20%), 2 to 3 repetitions and 2 to 4 min of rest were performed. Thereafter, a small increase in the load (5%) and 2 to 4 min of rest were carried out to reach the 1RM squat. The test was finished when the subjects failed to complete the squat, and the last successful attempt presents the 1RM back squat [14,15]. The intraclass correlation coefficient (ICC) for the pretest and posttest reliability was 0.93.

#### 2.3.4. Swimming Performance Measurements

Swimming performance measurements were established on the second day of tests also at 10.00 am. The 100 m front crawl performance and the 30 m leg flutter kick swimming were measured by two qualified timekeepers per stopwatch (SEIKO S120-4030, Tokyo, Japan) and noted in seconds. The starting signal was given by the timers, and diving start was performed in the 100 m front crawl test, whereas a push-off start was used in the 30 m leg kick swimming [1]. The pretest and posttest reliability for 100 m front crawl was ICC = 0.91, and for the 30 m leg kick swimming, ICC = 0.93.

**Figure 3:**
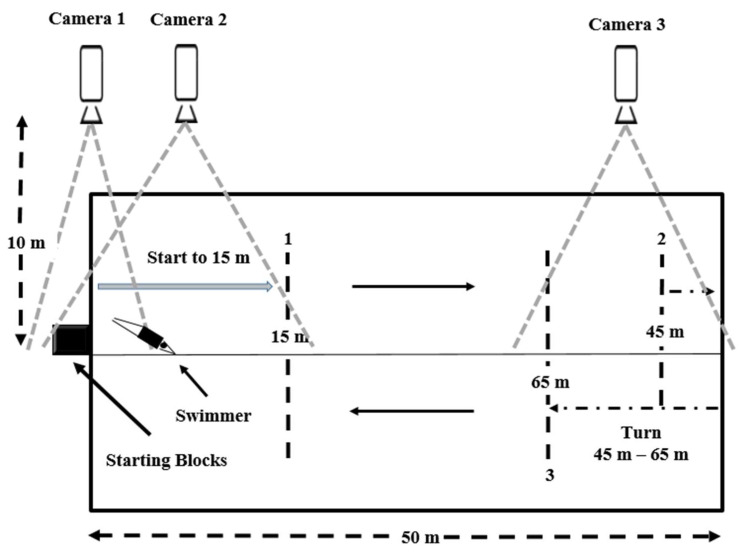
Set-up to analyze the start and turn performances.

To determine the start and turn performances, three cameras (SNC VB 603, Sony, Tokyo, Japan; *f* = 50 Hz, full HD, 1080 p) were set at well-defined points laterally to the swimming pool about 5 m above the water surface and about 10 m away from the swimming lane to film the 100 m front crawl test (Figure 3) [1]. A video analysis system (Kinovea, version 0.8.15, Joan Charmant & Contrib., Kinovea.org, accessed on 3 January 2022) was used to evaluate: (i) start performances: Block time (the time lag between the starting signal and the instant the swimmer’s feet left the block) and the 15 m time (the time lag between the starting signal and the swimmer’s head reaching the 15 m mark). (ii) turn performance: the total turn (the time lag between reaching the 45 m mark and the 15 m mark of the following split) [7,16]. The pretest and posttest reliability of start and turn performances were 0.89 ≤ ICC ≤ 0.95.

### 2.4. Statistical Analyses

SPSS 26.0 (SPSS Inc., Chicago, IL, USA) was used for statistical analysis. All data are presented as mean and SD, mean difference, partial difference in percentage, and 95% confidence interval. The baseline between-group differences were computed through independent sample *t*-tests. Normality and sphericity of the data were tested and confirmed using Shapiro–Wilk test and Mauchly test, respectively. Pre and post test reliability was assessed using the intraclass correlation coefficient (ICC_2,1_) [17]. The standard error of measurement (SEM) was determined by SD of the pretest × √(1-ICC) [18]. Minimum detectable change (MDC) of all dependent variables was derived using SEM × √2 × 1.96, and MDC % was defined as ((MDC/mean of pretest) × 100) [18]. Two-way ANOVA was used to determine the change of strength and swimming performance between pre and post test. The effect size (ES) was assessed by converting partial Eta-squared to Cohen’s d [19]. ES was classified as trivial (d < 0.25), small (0.25 ≤ d < 0.50), moderate (0.50 ≤ d < 1) and large (d ≥ 1) [20]. The level of significance was established at *p* ≤ 0.05.

## 3. Results

No significant difference in the baseline values between both groups in anthropometrics, swimming performance, and maximum strength test were shown (*p* > 0.05). Significant main effects of time, group, and time × group interaction were shown in all dependent variables (*p* < 0.05).

A significant improvement in 1RM back squat (14.94 ± 1.32%, *p* < 0.001, ES = 1.90 (large)) in EG was found while the 1RM back squat weight remained unchanged in CG (*p* > 0.05, Table 2).

In addition, significant improvement was found in 100 m front crawl (4.41 ± 1.39%, *p* = 0.001, ES = 1.83 (large)) and in 30 m leg kick swimming (5.84 ± 0.16%, *p* < 0.001, ES = 2.11 (large)) after 9 weeks of intervention period in the experimental group, but not in the control group (Table 2).

Also a significant improvement in all start performances (15 m time: 2.69 ± 0.18%, *p* < 0.001, ES = 2.77 (large); block time: 15.14 ± 1.06, *p* < 0.001, ES = 2.61 (large)) and in turn performance (total turn: 2.88 ± 0.08, *p* = 0.001, ES = 1.85 (large)) in the experimental group, while no significant changes in any of these parameters were observed in the control group (*p* > 0.05, Table 2).

## 4. Discussion

The aim of the present study was to investigate the effect of concurrent resistance training in water (parachute) and dry land on maximum lower body strength, leg kick, and swimming performances in competitive swimmers. The main results showed significant increases in strength and all swimming variables after nine weeks of combined resistance training, while the control groups did not have positive improvements in any of the selected variables.

Combined resistance training for nine weeks increased the 1RM back squat by 14.94 ± 1.32%. This improvement is in accordance with previous studies conducting dry land interventions between 8 and 10 weeks and intensity between 60% and 80% 1RM [2,21,22]. Garrido et al. [21] also showed that eight weeks of resistance dry land training that included leg extension (intensities between 50% and 75% of the 1RM), CMJ and CMJ box exercises with 2 to 3 sets and 5 to 8 repetitions improved the performance of the 6RM leg extension (55.6%) in young competitive swimmers (12.08 ± 0.76 years). Likewise, Kubo et al. [22] showed 11.3 ± 8.6% improvement in the 1RM half squat performance after 10 weeks of lower limb training (full squat and half squat exercises) in healthy male subjects (age = 20.9 years). In addition, Amara et al. [2] reported that nine weeks of different strength training load including leg extension exercise (high load: 5 to 6 sets and 3 to 5 repetitions; moderate load: 4 to 5 sets and 3 to 5 repetitions; low load: 3 to 4 sets and 3 to 5 repetitions) with intensity between 85% and 95% 1RM leg extension improved the 1RM leg extension (9.87% to 19.82%) in male competitive swimmers. Hence, 8–10 weeks of training at 60–80% of 1RM yields improvements in maximum and power strength of lower limb [2,21,22]. As such, the dry land program design for this research is as effective as others reported in the literature.

According to the previous literature, this study is the first investigation that studied the effect of combined resistance training on leg kick swimming performance; for this reason, it is challenging to benchmark these results against previous findings. Only, Konstantaki et al. [23] noted that training during six weeks based on leg kick training (3 sessions per week) could improve the performance of the 200 m leg kick swimming (6 ± 2%) in male competitive swimmers. Furthermore, the concurrent aquatic and lower limb resistance training improved the 30 m leg kick swimming performance (5.84 ± 0.16%), which is probably caused by the improvement in maximum lower body strength as suggested by the increased 1-RM squat results. More specifically, the transfer of force gain from the lower limb to the leg kick swimming was clearly evident in the EG (1.22 s) better than in the CG (0.22 s) with a difference of ≈1 s, and this explains the effective role of resistance lower limb training in improving the performance of the leg kick swimming in the water.

The 100 m front crawl times increased by approximately 2.63 s (4.41 ± 1.39%) after the concurrent resistance training. In fact, the increase in the maximum strength and the leg kick swimming performance in the EG (9.45 kg, 1.22 s, respectively) compared in the CG (1.46 kg, 0.22 s, respectively) could explain that the swimmers of EG make suitable use of the transfer of gain of force to improve the 100 m swimming performance (2.63 s) compared in CG (0.28 s) with a difference of ≈ 2.35 s. This present investigation is a complement to the previous study developed by Amara et al. [1]. Whereas Amara et al. [1] revealed an improvement of 25 m front crawl arm-pull (1.05 s) due to the transfer of force gain after the increase in maximum upper limb performance (5.45 kg), and subsequently, the sprint swimming velocity was optimized (0.16 ms^−1^). On the other hand, periodization and training planning plays an important role in further improving front crawl performance. Whereas, 8 to 9 weeks of concurrent resistance training during the preparatory phase could be sufficient to cope with the new imposed aquatic and in dry land training load and subsequently the improvement sprint swimming performance (4.22% to 6.82%) [1,3].

A start and turn performance improvement (2.96% to 15.14%) was shown after 9 weeks of concurrent resistance training, in which the contribution of the legs is predominant [5]. An increase in the force of pushing by the legs at the starting block level (start) and at the wall level (turn) due to the improvement in the maximum force of the lower limb can explain these present results [24,25]. More specifically, the neuromuscular adaptations represented by the learning and coordination of the back squat exercise during the 9 weeks of training, the competitive level of the swimmers, which could favor the specificity of the adaptation to the training, and the transfer of gain of strength may be indirect evidence that justifies the results obtained at the start and turn level [26]. In addition, this study does not present any specific technical start and turn training; therefore, there is no change in the start and turn technique in the two groups. This could confirm the important contribution of maximum lower limb strength in the optimization of start and turn performance.

To sum up, 9 weeks of concurrent training included sets of aquatic resistance and dry land training with 1 to 2 sessions per week, 2 to 3 sets, 6 to 10 repetitions, and intensity between 60% and 80% of 1RM could improve the maximum lower limb strength and ultimately the optimization of swimming performance.

This study has some methodological limitations that warrant discussion. Perhaps the combined resistance training is a limitation because we do not know exactly what the partial contribution of each type of training (dry land vs. in water) is to strength improvement. The small sample size could also be a limiting factor in this study. In addition, the present study only includes male swimmers. Future studies must include female counterparts. Added to that, it is required to reproduce the combined resistance training reported here in other swim strokes (breaststroke, butterfly, and backstroke) and competitive levels. The effect of concurrent resistance training on certain physiological variables (cardiorespiratory adaptation to physical exertion) may also be a future field of study.

## 5. Conclusions

This study showed that 9 weeks of concurrent resistance training can improve the maximum lower limb strength. In addition, our findings showed that suitable transfer of strength gain to leg kick swimming, start and turn performance after the concurrent resistance training ultimately improved the 100 m front crawl performance. Concurrent resistance training included dry land exercises (back squat, CMJ, and CMJ box) with 1 to 2 sessions per week, 2 to 3 sets, 6 to 10 repetitions, and intensity between 60% and 80% of 1RM and aquatic resistance training with water parachute is an effective strategy that can be incorporated by coaches and swimmers into their training programs to improve sprint swimming performance in competitive swimmers.

## Figures and Tables

**Table 1:** Detailed resistance training conducted by the experimental group.

Week (Sessions)	Exercises	Sets × Repetition × Intensity; Recovery between Sets and Exercises (3 min)
1 (1/2)	Back squat	2 × 10 × 60% 1RM
	CMJ	2 × 6
	CMJ box	2 × 6
2 (3/4)	Back squat	2 × 10 × 65% 1RM
	CMJ	2 × 8
	CMJ box	2 × 8
3 (5/6)	Back squat	2 × 10 × 70% 1RM
	CMJ	2 × 10
	CMJ box	2 × 10
4 (7/8)	Back squat	2 × 8 × 75% 1RM
	CMJ	3 × 8
	CMJ box	3 × 8
5 (9/10)	Back squat	3 × 6 × 80% 1RM
	CMJ	3 × 10
	CMJ box	3 × 10
6 (11/12)	Back squat	2 × 8 × 75% 1RM
	CMJ	3 × 6
	CMJ box	3 × 6
7 (13)	Back squat	3 × 6 × 80% 1RM
	CMJ	3 × 10
	CMJ box	3 × 10
8 (14)	Back squat	3 × 8 × 75% 1RM
	CMJ	3 × 8
	CMJ box	3 × 8
9 (15)	Back squat	3 × 8 × 70% 1RM
	CMJ	3 × 6
	CMJ box	3 × 6

1RM: one repetition maximum; CMJ: countermovement jump; CMJ box: countermovement jump box.

**Table 2:** Changes in the maximum lower body strength, swimming, start and turn performances between the pre and post test in the experimental (EG) and control groups.

Variables	Groups	Pre Test	Post Test	*p*-Value	Effect (95% CI)	Δ (%)	MDC (%)	Effect Size
1RM back squat (kg)	EG	63.46 ± 4.99	72.91 ± 5.45	<0.001	5.27 (1.88 to 8.66)	14.94 ± 1.32	3.89 (6.19)	1.90 (large)
	CG	62.18 ± 5.72	63.64 ± 6.04	0.568		2.32 ± 0.73		0.46 (small)
100 m front crawl (s)	EG	59.56 ± 1.50	56.93 ± 1.51	0.001	−1.10 (−2.03 to −0.17)	4.41 ± 1.39	1.25 (2.10)	1.83 (large)
	CG	59.49 ± 1.55	59.21 ± 1.55	0.678		0.47 ± 0.02		0.19 (trivial)
30 m Leg kick swimming (s)	EG	20.97 ± 0.61	19.75 ± 0.61	<0.001	−0.65 (−1.02 to −0.28)	5.84 ± 0.16	0.44 (2.09)	2.11 (large)
	CG	21.12 ± 0.60	20.90 ± 0.60	0.398		1.04 ± 0.05		0.39 (small)
Time 15 m (s)	EG	6.65 ± 0.07	6.47 ± 0.07	<0.001	−0.07 (−0.11 to −0.03)	2.69 ± 0.18	0.06 (0.90)	2.77 (large)
	CG	6.65 ±0.07	6.61 ± 0.06	0.159		0.60 ± 0.22		0.66 (moderate)
Block time (s)	EG	0.78 ± 0.05	0.66 ± 0.05	<0.001	−0.05 (−0.08 to −0.02)	15.14 ± 1.06	0.06 (7.69)	2.61 (large)
	CG	0.78 ± 0.05	0.75 ± 0.05	0.216		3.39 ±0.65		0.57 (moderate)
Total turn (s)	EG	11.48 ± 0.19	11.15 ± 0.18	0.001	−0.14 (−0.25 to −0.03	2.88 ± 0.08	0.11 (0.96)	1.85 (large)
	CG	11.48 ± 0.17	11.42 ± 0.17	0.434		0.51 ± 0.04		0.36 (small)

MDC: minimal detectable change; Δ: delta change (pre test to post test).

## Data Availability

The data presented in this study are available on reasonable re-quest from the corresponding author.
